# Diagnostic value of procalcitonin and C reactive protein for infection and sepsis in elderly patients

**DOI:** 10.3906/sag-2007-268

**Published:** 2021-10-21

**Authors:** Çiler ZİNCİRCİOĞLU, Kazım ROLLAS, Işıl Köse GÜLDOĞAN, Aykut SARITAŞ, Hüseyin ÖZKARAKAŞ, Gürsel ERSAN, Nimet ŞENOĞLU

**Affiliations:** 1 Department of Anesthesiology and Reanimation, Intensive Care Unit, SBU Tepecik Training and Research Hospital, İzmir Turkey; 2 Department of Anesthesiology and Reanimation, Intensive Care Unit, Bozyaka Training and Research Hospital, İzmir Turkey; 3 Department of Infectious Diseases and Clinical Microbiology Clinic, SBU Tepecik Training and Research Hospital, İzmir Turkey

**Keywords:** Aged, procalcitonin, C-reactive protein, infections, sepsis

## Abstract

**Background/aim:**

Biomarkers are useful for diagnosing infection and sepsis in adults, but data are limited in elderly patients. Furthermore, clinical symptoms of infection in elderly patients are usually atypical or unclear. We aimed to assess the usefulness of PCT, CRP, and WBC in distinguishing elderly patients infected with sepsis from infected without sepsis and those with no-infection. We also aimed to find a cut-off value for diagnosing sepsis and infection without sepsis in elderly critically ill patients.

**Materials and methods:**

In this single-center and prospective observational study, patients older than 65 years were enrolled. Serum levels of PCT, CRP, and WBC were measured within 24 h. Patients were allocated into sepsis (S), infected without sepsis (IWS), and no-infection (NI) groups. Data were analyzed with Mann–Whitney U test and Kruskal–Wallis test.

**Results:**

We analyzed 188 patients with a mean age of 77.05 ± 7.4 in the study; 95 (50.5%) of them were women. Sixty-four (34%) of whom were classified as IWS, 29 (15%) as S, and 95 (50.5%) as NI group. There were significant differences in the PCT, CRP levels between the IWS and NI, S and NI (p < 0.001, p < 0.001, p < 0.001, p < 0.01, respectively). The PCT levels were significantly different when the NI group was compared to IWS (p < 0.001) and S (p < 0.001) groups. The CRP levels were also different when the NI group was compared to both IWS (p < 0.001) and S (p < 0.001). The PCT cut-off values were 0.485 μ/L and 1.245 μg/L for the discrimination of patients with IWS and S, respectively. The cut-off values of CRP level were 59.45 mg/L and 57.50 mg/L for infected without sepsis and sepsis, respectively.

**Conclusion:**

PCT was found to be a more valuable marker than CRP and WBC for the discrimination of elderly patients with infected without sepsis and sepsis.

## 1. Introduction 

The proportion of elderly patients admitted to the intensive care unit (ICU) has gradually increased throughout the past decades as a result of demographic alterations (1). Bacterial infection is one of the leading causes of hospitalization and death in the elderly population (2). Although malnutrition and age-related physiological and anatomical changes are important causes of increased susceptibility to infectious diseases in the elderly, the leading cause is decreased immune function (3). Because of this deficient immune response in elderly patients, bacterial infections present with atypical features, such as low or no fever and irregular leukocytosis, which challenge the physician in their diagnosis (4). However, early and accurate diagnosis of bacterial infection and appropriate antimicrobial treatment is associated with better outcomes (5). Several studies and meta-analyses have shown that procalcitonin (PCT) and other inflammatory blood markers [C-reactive protein (CRP) and white blood cells (WBC)] are reliable diagnostic markers of bacterial infection in the adult population (6–8). However, the values of biomarkers for diagnosing bacterial infection and sepsis have not been sufficiently determined in elderly patients (9–11). 

The purpose of our study to investigate the discriminative value of serum PCT, CRP, and WBC levels in distinguishing sepsis from infected without sepsis and no-infection, as well as to determine the optimal cut-off values of PCT and CRP for infection and sepsis in elderly critically ill patients. 

## 2. Materials and methods 

This observational, single-center, prospective study was performed between January 1st, 2017, and December 1st, 2019. The local ethics committee approved the study (No. 31829978-050.01.04), and written informed consent was obtained from all patients or the next of kin. 

ICU patients older than age 65 years were enrolled. Patients were excluded if they had the following conditions: death within 72 h after ICU admission, antibiotic treatment for more than 24 h before admission, chemotherapy within 90 days, chronic renal failure, immunosuppressant therapy, severe trauma or operation, the need for a vasopressor, or septic shock at admission to the ICU.

The age, sex, and comorbidities of the patients were recorded. The Simplified Acute Physiology Score (SAPS II) and the Sequential Organ Failure Assessment (SOFA) score were calculated. Blood samples for biomarker measurements (PCT, CRP, WBC) were taken within 24 h of ICU admission. 

Medical records of the patients were reviewed by an infectious disease specialist, and patients were allocated into three groups at the end of the first 72 h of ICU admission: infected without sepsis (IWS), with sepsis (S), and no-infection (NI).  

The infection types were defined considering clinical symptoms (body temperature > 38 °C or <36 °C, purulent sputum or urine, chest X-ray or ultrasound exams, computed tomography, laboratory tests, urine and blood culture results according to recommendations from the Centers for Disease Control and Prevention that define specific types of infection (http://www. cdc.gov/nhsn/pdfs/pscmanual/17pscnosinfdef_current.pdf (accessed 30 July 2019)). 

Sepsis was defined as life-threatening organ dysfunction caused by a host response to infection (12). Organ dysfunction was defined as an increase of 2 points on the SOFA score (12). 

### 2.1. Measurement of biomarkers

Serum PCT levels were measured with the electrochemiluminescence immunoassay method using a Roche Cobas E411 device at a reference range of 0.04–0.1 µg/L. Serum CRP levels were assayed via the nephelometric method using the Beckman Array 360 System at a reference range of 0–5 mg/L. The WBC count was determined using a hematological cell counter (LH 780 Analyzer, Beckman Coulter Inc., Miami, FL, USA) at a reference range of 4.2–10.6 × 10^3^/µL.

### 2.2. Statistical analysis

Means ± standard deviations were calculated for continuous variables. Normality was assessed using the Kolmogorov–Smirnov test. Nonnormally distributed variables were expressed as medians and as minimums and maximums. Categorical variables were presented as percentages. The Mann–Whitney test or the Kruskal–Wallis test was used, and the corrections were made Bonferroni test, when appropriate, for the comparison of continuous variables. Categorical variables were assessed using the chi-square test. Receiver operating characteristic (ROC) curves were plotted for PCT, CRP, and WBC levels to identify infection and sepsis. The areas under the ROC curve (AUCs) were calculated. AUC values were reported with 95% confidence intervals (95% CIs), and p < 0.05 was considered significant. Statistical analysis was performed with SPSS version 22.0 (SPSS Inc., Chicago, IL, USA).

## 3.Results

### 3.1. Study population and baseline characteristics 

A total of 1316 patients was admitted to our ICU 590 patients older than age 65 years were screened. Of these 590 patients, 402 were excluded according to the following conditions: death within 72 h after ICU admission (n = 25), history of recent antibiotic treatment for more than 24 h before admission (n = 258), septic shock, and/or need for a vasopressor at ICU admission (n = 80), chronic renal failure (n = 29), or severe trauma (n = 10). Thus, 188 patients with a mean age of 77.05 ± 7.4 and 95 (50.5%) of whom were women were included in this study for the final analysis. Sixty-four patients (34%) were assigned to IWS; 29 (15%) to S; and 95 (50.5%) to NI. Table 1 shows the baseline characteristics of the patients. Significant differences existed in the SAPS II and the SOFA scores between the IWS and NI (p < 0.001 for each score), S and NI (p < 0.001 for each score), and IWS and S groups (p < 0.01 for each score). Significant differences were measured in creatinine values between the S and NI (p < 0.001) and IWS and S (p < 0.001) groups but not between the IWS and NI groups (p = 0.95). 

**Table 1:** General characteristics of elderly patients.

	IWS group(n = 64)	S group (n = 29)	NI group (n = 95)	p value*
Age (years) mean ± SD	76.7 ± 7.4	75.9 ± 8.3	77.6 ± 7.2	0.41
Female n (%)	29 (45)	17 (58)	49 (51)	0.46
SAPS score	66 (28–133)	81 (21–133)	41 (21–98)	<0.001
SOFA score	3.5 (2–8)	6 (3–10)	3 (1–5)	<0.001
Underlying diseases (n)				
HF	9	5	26	0.11
IHD	14	4	17	0.85
DM	3	0	6	0.38
COPD	6	1	10	0.50
Neurological disease	12	9	8	<0.01
Cancer	8	4	11	0.94
Two or more underlying diseases	11	6	17	0.70
Peak temperature (°C) (min-max)	37.5 (37.1–38.3)	37.9 (37.5–38.3)	37.0(36.6–37.4)	<0.001
Creatin (mg/dL) (min-max)	1.2 (0.40–3.20)	1.8 (0.8–3.0)	1.1 (0.40–4.20)	<0.001

*p values show the results of the Kruskal–Wallis test for continuous variables and results of the chi-square test for categorical variables, which were conducted to compare the three groups. Pairwise comparisons by Mann–Whitney U test and chi-square test were shown in the results section of the text and summarized below: Significant differences were found in SAPS II and the SOFA scores between the IWS and NI (p < 0.001 for each score), S and NI (p < 0.001 for each score), and IWS and S groups (p < 0.01 for each score). Significant differences were found in creatinine values between the S and NI (p < 0.001), IWS and S (p < 0.001) groups, IWS and NI groups (p = 0.95). Compared to patients in the NI group, patients in the S group had more neurological diseases (p < 0.01).

Compared to patients in the NI group, patients in the S group had more neurological diseases (p < 0.01). The number of associated neurological diseases was not significantly different between the IWS and S groups (p = 0.19) or the IWS and NI groups (p = 0.06). No significant differences were observed among the three groups in terms of the existence of other underlying diseases. In the IWS group, pneumonia (n = 41; 64.0 %) was the most common infection type, followed by urinary tract infection (UTI; n = 14; 22.0%) and then skin and soft tissue infection (n = 9; 14.0%). The most common causes of sepsis were pneumonia (n = 13; 45%), UTI (n = 11; 38%), biliary tract infection (n = 3; 10%), and skin and soft tissue infection (n = 2; 7%). 

 3.2. Comparison of WBC, PCT, and CRP levels among IWS, S, and NI groups 

In the IWS group (n = 64), the median (minimum-maximum) PCT, CRP, and WBC values were 2.5 µg/L (0.03–48.04), 68.7 (8.2–158.8), and 15.9 (4.2–64.8), respectively. In the S group (n = 29), the median (minimum-maximum) PCT, CRP, and WBC values were 18.6 µg/L (0.69–93.6), 88.3 mg/L (13.6–184.2), and 19.7 (3.5–46.1), respectively. In the NI group (n = 95), the median (minimum-maximum) PCT, CRP, and WBC values were 0.09 µg/L (0.01–2.6), 10 mg/L (0.9–72.0), and 25.5 × 10^3^/µL (1.4–140.1), respectively (Table 2).

**Table 2:** Comparison of PCT, WBC, and CRP levels in patients with IWS, S, and NI group.

	IWS group (n = 64)	S group (n = 29)	NI group(n = 95)	p value*
PCT(µg/L)	2.52 (0.03–48.04)	18.6 (0.69–93.6)	0.09 (0.01–2.6)	<0.001
CRP (mg/L)	68.8 (8.2–158.8)	88.3 (13.6–184.2)	25.5 (1.4–140.1)	<0.001
WBC(× 103/µL)	15.9 (4.2–64.8)	19.7 (3.5–46.1)	10 (0.9–72.0)	<0.001

*p values show the Kruskal–Wallis test results for continuous variables that were conducted to compare the three groups. Pairwise comparisons by Mann–Whitney U test were shown in the results section of the text and summarized below: There were significant differences in the PCT levels between the IWS and NI, S and NI, and IWS and S groups (p < 0. 001 for each group). Significant differences were noted in the CRP levels between the IWS and NI groups and the S and NI groups (p < 0.001 for each comparison) but not between the IWS and S groups (p = 0.80). The WBC levels were significantly different between the IWS and NI groups and between the S and NI groups (p < 0.001 for each difference) but not between the IWS and S groups (p = 0.07).

There were significant differences in the PCT levels between the IWS and NI, S and NI, and IWS and S groups (p < 0. 001 for each group). Significant differences were noted in the CRP levels between the IWS and NI groups and the S and NI groups (p < 0.001 for each comparison) but not between the IWS and S groups (p = 0.80). The WBC levels were significantly different between the IWS and NI groups and between the S and NI groups (p < 0.001 for each difference) but not between the IWS and S groups (p = 0.07) (Table 3).

**Table 3:** Comparison of PCT, WBC, and CRP levels in patients with IWS and S group.

	IWS group (n = 64)	S group (n = 29)	p value*
PCT (µg/L)	2.52 (0.03–48.04)	18.6 (0.69–93.6)	<0.001
CRP (mg/L)	68.75 (8.2–158.8)	88 (13.6–184.2)	0.80
WBC (× 103/µL)	15.95 (4.2–64.8)	19.7 (3.5–46.1)	0.07

### 3.3. Diagnostic performance of PCT, CRP, and WBC

#### 3.3.1. Infected without sepsis

**Figure 1:**
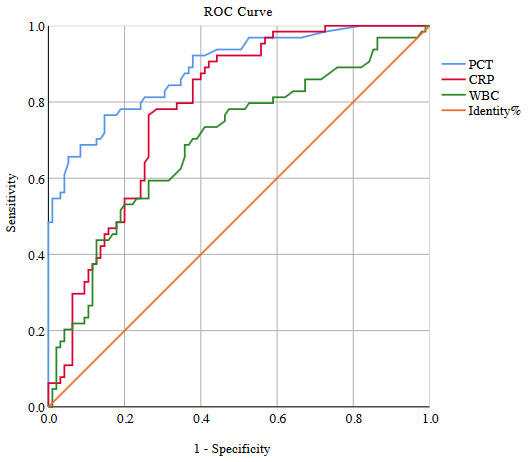
ROC curve analysis of the PCT, CRP, WBC to identify infected without sepsis. PCT, procalcitonin; CRP, C-reactive protein; WBC, white-blood-cell.

ROC curves were performed for PCT, CRP, and WBC levels to identify infections in the IWS group, and the AUCs were calculated (Figure 1). The PCT level was a good marker for the discrimination of patients with IWS; its cut-off point was 0.485 µg/L (sensitivity, 76.56%; CI: 64.31–86.25; specificity, 85.26%; CI: 76.51–91.70). The AUC for discriminating patients with IWS according to PCT was 0.886 (95% CI: 0.84–0.94; p < 0.001). The cut-off point of the CRP level for the discrimination of patients with IWS was 59.45 mg/L (sensitivity, 76.56%; CI: 64.31–86.25, specificity 73.68%; CI: 63.65–82.19), and the AUC for identification of IWS according to CRP was 0.787 (95% CI: 0.72–0.86; p < 0.001). The cut-off point of the WBC level for the discrimination of patients with IWS was 15.40 × 10^3^/µL (sensitivity, 53.12%; CI: 40.23–65.72; specificity, 80.0%, CI: 70.54–87.51), and the AUC for identification of IWS according to WBC was 0.695 (95% CI: 0.61–0.78; p < 0.001; Table 4).

**Table 4:** The cut-off point of PCT, WBC and CRP levels for discrimination of patients with IWS.

Cut-off point	Sensitivity(95% CI)	Specificity(95% CI)	PPV (95% CI)	NPV(95% CI)	AUC(95% CI)	p*
PCT ≥ 0.485 µg/L	76.56 %(64.31–86.25)	85.26%(76.51–91.70)	77.78%(67.93–85.26)	84.38%(77.48–89.45)	0.886(0.84–0.94)	<0.001
CRP ≥ 59.45 mg/L	76.56%(64.31–86.25)	73.68%(63.65–82.19)	62.22%(57.69–73.80)	82.35%(74.68–88.07)	0.787(0.72–0.86)	<0.001
WBC≥ 15.40 × 103/uL	53.12%(40.23–65.72)	80.0%(70.54–87.51)	64.15%(52.96–73.99)	71.70%(65.70–77.01)	0.695(0.61–0.78)	<0.001

*Higher values of CRP, PCT, and WBC indicate stronger evidence for Infected without sepsis. PCT, procalcitonin; CRP, C-reactive protein; WBC, white blood cells; IWS, infected without sepsis; CI, confidence interval; PPV, positive predictive value; NPV, negative predictive value; AUC, area under curve.

#### 3.3.2. Sepsis

**Figure 2:**
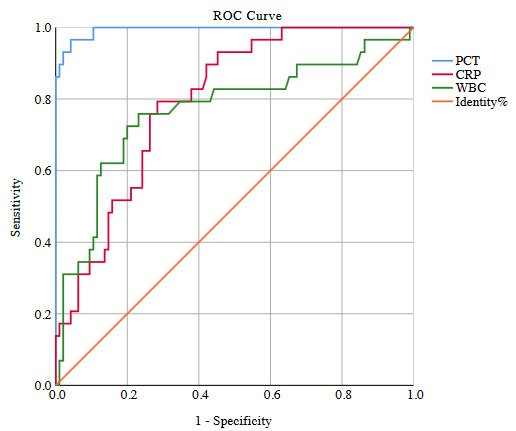
ROC curve analysis of the PCT, CRP, WBC to identify sepsis. PCT, procalcitonin; CRP, C-reactive protein; WBC, white-blood-cell.

ROC curves were performed for PCT, CRP, and WBC levels to identify sepsis in the S group, and AUCs were calculated (Figure 2). The PCT level was a good marker for the discrimination of patients with S; its cut-off point was 1.245 µg/L (sensitivity, 96.55%; CI: 82.24–99.91; specificity, 95.79%; CI: 89.57–98.84). The AUC for discriminating patients with S according to PCT was 0.994 (95% CI: 0.99–1.0; p < 0.001). The cut-off point of the CRP level for the discrimination of patients with S was 57.50 mg/L (sensitivity, 79.31%; CI: 60.28–92.01; specificity, 71.58%; CI: 61.40–80.36), and the AUC for identification of S according to CRP was 0.795 (95% CI: 0.71–0.88; p < 0.001). The cut-off point of the WBC level for the discrimination of patients with S was 14.65 ×10^3^/µL (sensitivity, 75.86%; CI: 56.46–89.70; specificity, 76.84%; CI: 67.06–84.88), and the AUC for identification of S according to WBC was 0.768 (95% CI: 0.66–0.88; p < 0.001; Table 5). 

**Table 5:** The cut-off point of PCT, WBC, and CRP levels for discrimination patients with S.

Cut-off point	Sensitivity	Specificity	PPV	NPV	AUC(95% CI)	p*
PCT ≥1.245µg/L	96.55%(82.24–99.91)	95.79%(89.57–98.84)	87.50%(72.80–94.82)	98.91%(92.99–99.84)	0.994(0.99–1.0)	<0.001
CRP≥ 57.50mg/L	79.31%(60.28–92.01)	71.58%(61.40–80.36)	46.00%(37.06–55.21)	91.89%(84.61–95.90)	0.795(0.71–0.88)	<0.001
WBC≥ 14.65 × 109/uL	75.86%(56.46–89.70)	76.84%(67.06–84.88)	50.0%(39.65–60.35)	91.25%(84.42–95.25)	0.768(0.66–0.88)	<0.001

*Higher values of CRP, PCT, and WBC indicate stronger evidence for sepsis. PCT, procalcitonin; CRP, C-reactive protein; WBC, white blood cells; S, sepsis; CI, confidence interval; PPV, positive predictive value; NPV, negative predictive value; AUC, area under curve.

## 4. Discussion

In this prospective, observational study, we investigated the diagnostic value and optimal cut-off points of serum PCT and CRP levels for sepsis and infection without sepsis in elderly critically ill patients. Our results showed that PCT is a more valuable marker for the diagnosis of IWS (AUC = 0.886, p < 0.001) and S (AUC=0.994, p < 0.001) when accompanied by ROC analysis. On the other hand, we found that PCT is an accurate marker for distinguishing sepsis from IWS, while CRP and WBC are not. We also found cut-off levels of PCT (IWS, 0.485 µg/L; S, 1.245 µg/L) and CRP (IWS, 59.45 mg/L; S, 57.50 mg/L) for diagnosing IWS and S in elderly patients were higher than the current standard cut-off levels.

Diagnosis of infection in elderly patients is quite complicated by age-related changes, absence of symptoms, such as fever, and comorbidities that make physical evaluation difficult (13). Although acute phase reactants, such as leukocyte count, CRP, and PCT, are considered in the diagnosis of sepsis and bacterial infections in elderly patients, their reliability and the optimal cut-off points in the diagnosis of IWS and S have not been sufficiently determined (9–11). 

Similar to our study, numerous studies have confirmed that PCT levels have a higher specificity than CRP levels for bacterial infections (9, 14–17). For instance, Lee et al. (9) found that PCT is both specific and sensitive in the diagnosis of infection in elderly patients when leukocytosis is specific (specificity, 0.86) but poorly sensitive (sensitivity, 0.26), and they noted that CRP is highly sensitive (sensitivity, 0.91) but nonspecific (specificity, 0.36). In another study, Lin et al. (17) found that PCT levels were more effective than CRP levels and WBC count at diagnosing bacterial infection in patients older than age 65 years with diabetes. According to the results reported by Lin et al. (17), optimal PCT cut-off points for diagnosing lung infection, UTI, and skin and soft tissue infection are, respectively, 0.73 µg/L, 1.48 µg/L, and 0.73 µg/L —all higher than the standard value (0.25 µg/L). 

Despite the predominantly positive results regarding the superiority of PCT use in the diagnosis of systemic infections, some studies show that CRP is more useful than PCT (18–21). In a large retrospective study, serum high-sensitivity CRP (hs-CRP) level (cut-off value, 61 mg/L) at admission was more useful than PCT in the diagnosis of pneumonia in hospitalized elderly patients (18). In another study conducted in an emergency department, which included patients older than age 65 years, the CRP and erythrocyte sedimentation rate were more reliable markers for differentiating sepsis from SIRS compared with PCT, WBC, and interleukin-6 levels (19). Stucker et al. (20) demonstrated that increased CRP (≥3 mg/L) in patients older than age 75 years in a geriatric ward was an independent predictor for the presence of acute infection. However, the authors did not find a significant relationship between infection and elevated serum PCT (20). Zhang et al. (21) found that hs-CRP was not inferior to PCT for the diagnosis of sepsis and septic shock in patients who were older than age 85 years. In this study, the optimum cut-off values of serum PCT and hs-CRP for the diagnosis of sepsis were 0.45 µg/L and 74.2 mg/L, respectively (21).  Our results showed PCT levels for diagnosing IWS and S in this population (IWS, 0.485 µg/L; S, 1.245 µg/L) was higher than the standard value (0.04–0.1 µg/L). Also, the optimum cut-off points of CRP levels for diagnosing IWS and S in this population were higher (IWS, 59.45 mg/L; S, 57.50 mg/L) compared to the standard value (0–5 mg/L).

Although many meta-analyses have investigated the diagnostic reliability of PCT and CRP in sepsis and local infection, there is no consensus for proposing a widely accepted cut-off value for these biomarkers (8, 9,22). Heterogeneity exists because of differences in cut-off values of PCT and CRP used in studies enrolling elderly patients (9,10,23). In their meta-analysis, Tan et al. (8) found that the optimal cut-off values of PCT and CRP showed heterogeneity between 0.76  to 6.03 µg/L and between 12.00  to 90.00 mg/L, respectively, in adult patients with sepsis. Liu et al. (24) found that the CRP cut-off of 60 mg/L had the best combination of sensitivity (80.7%) and specificity (96.0%). Another study found that the best cut-off value for PCT in older patients was 1.4 µg/L for the diagnosis of sepsis, similar to the cut-off result in our study (25). Patients with chronic kidney disease have higher PCT levels than the normal baseline, regardless of whether they receive renal replacement therapy or not (26). In our study, patients with chronic kidney disease were excluded. However, PCT clearance varies because of the decrease in the glomerular filtration rate caused by aging (27,28); this variation may explain why the PCT cut-off values in our study were higher than the standard values in the elderly population.

We noted in our study that leukocytosis was a moderately sensitive marker for IWS and S. Previous studies also found that WBC count was moderately useful (19,29) or useless (23) for ruling in or out bacteremia.

There are some limitations to be considered in our study. First, our study does not have adult patient control groups, so whether the values in our study are related to age or not is still a question to be answered. Our results indicate the values seen in elderly patients, but we cannot specify these values as related to age as we did not compare our patients with an adult patient group to evaluate differences in CRP and PCT kinetics related to age. Second, we did not obtain longitudinal data on dynamic changing trends in PCT, CRP, and WBC levels. Last, we conducted a single-center study; multicenter studies are needed for more precise results.

 In conclusion, according to the results of our study, including elderly critically ill patients, the diagnostic accuracy of PCT for sepsis and infected without sepsis is higher than that of CRP and WBC. We found that PCT is a more accurate marker for differentiating infected without sepsis and sepsis while CRP and WBC are not. More studies are needed to determine the PCT and CRP kinetics in elderly patients and identify the optimal cut-off point for PCT levels at different stages of infection. 

## Informed consent

The local ethics committee approved the study (No. 31829978-050.01.04), and written informed consent was obtained from all patients or the next of kin.
